# Continuous-Flow Laboratory SAXS for In Situ Determination of the Impact of Hydrophilic Block Length on Spherical Nano-Object Formation during Polymerization-Induced Self-Assembly

**DOI:** 10.1021/acs.macromol.3c00585

**Published:** 2023-08-04

**Authors:** Jonathan D. Guild, Stephen T. Knox, Sam B. Burholt, Eleanor.M. Hilton, Nicholas J. Terrill, Sven L.M. Schroeder, Nicholas J. Warren

**Affiliations:** † School of Chemical and Processing Engineering, University of Leeds, Woodhouse, Leeds LS2 9JT, U.K.; ‡ Diamond House, Harwell Science and Innovation Campus, Diamond Light Source, Didcot OX11 0DE, U.K.

## Abstract

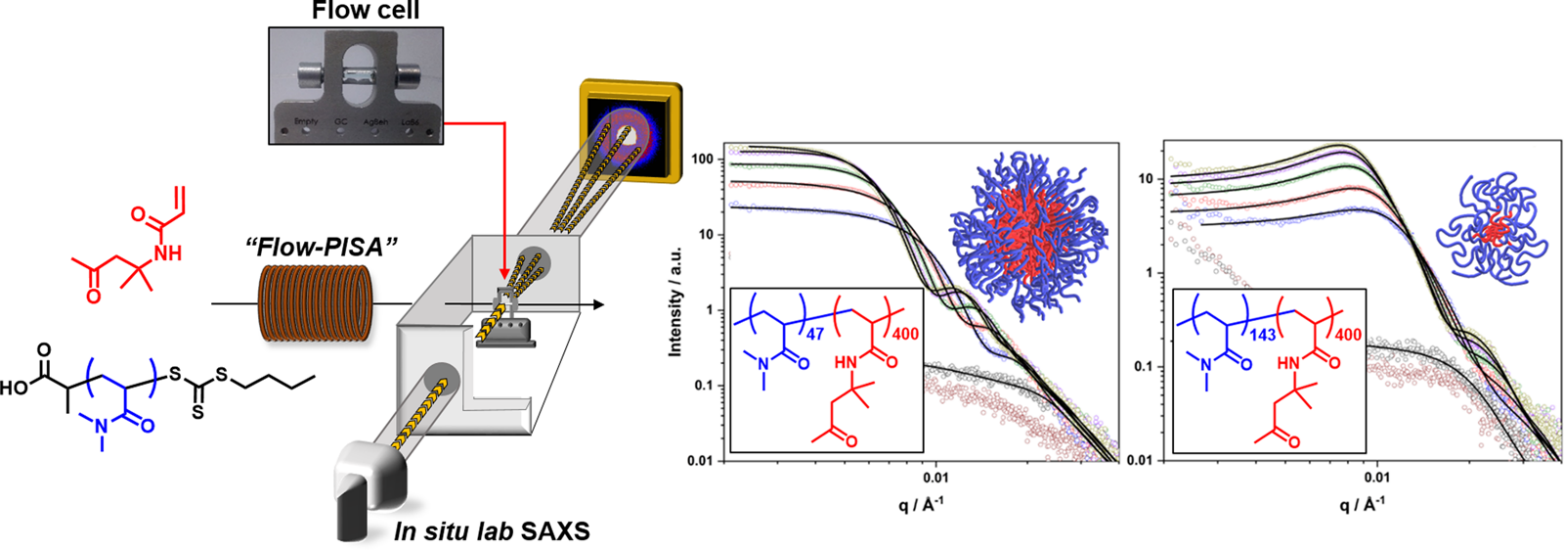

In situ small-angle X-ray scattering (SAXS) is a powerful technique for characterizing block-copolymer nano-object formation during polymerization-induced self-assembly. To work effectively in situ, it requires high intensity X-rays which enable the short acquisition times required for real-time measurements. However, routine access to synchrotron X-ray sources is expensive and highly competitive. Flow reactors provide an opportunity to obtain temporal resolution by operating at a consistent flow rate. Here, we equip a flow-reactor with an X-ray transparent flow-cell at the outlet which facilitates the use of a low-flux laboratory SAXS instrument for in situ monitoring. The formation and morphological evolution of spherical block copolymer nano-objects was characterized during reversible addition fragmentation chain transfer polymerization of diacetone acrylamide in the presence of a series of poly(dimethylacrylamide) (PDMAm) macromolecular chain transfer agents with varying degrees of polymerization. SAXS analysis indicated that during the polymerization, highly solvated, loosely defined aggregates form after approximately 100 s, followed by expulsion of solvent to form well-defined spherical particles with PDAAm cores and PDMAm stabilizer chains, which then grow as the polymerization proceeds. Analysis also indicates that the aggregation number (*N*_agg_) increases during the reaction, likely due to collisions between swollen, growing nanoparticles. In situ SAXS conducted on PISA syntheses using different PDMAm DPs indicated a varying conformation of the chains in the particle cores, from collapsed chains for PDMAm_47_ to extended chains for PDMAm_143_. At high conversion, the final *N*_agg_ decreased as a function of increasing PDMAm DP, indicating increased steric stabilization afforded by the longer chains which is reflected by a decrease in both core diameter (from SAXS) and hydrodynamic diameter (from DLS) for a constant core DP of 400.

## Introduction

Polymerization-induced self-assembly (PISA) provides a convenient method for preparing block copolymer nano-objects with a range of morphologies.^1−6^ Due to this ability to tailor the size and morphology of these nano-objects, they have been proposed for a diverse range of applications including as Pickering emulsifiers,^7^ drug delivery vectors,^8−11^ or in porous membranes.^12^

PISA is typically conducted by reversible addition fragmentation chain transfer (RAFT) polymerization of a soluble monomer in the presence of a soluble macromolecular chain transfer agent (macro-CTA).^1,6,13−17^ During the early stages of the process, short, soluble diblock copolymer chains are formed, but as the second block reaches a critical degree of polymerization (DP) the growing block becomes insoluble, resulting in amphiphilic chains which undergo self-assembly to form nano-objects. These can be in the form of spheres, worms, or vesicles depending on factors such as concentration, relative volume fractions of the blocks and temperature.^18−23^

In water, a large array of formulations have been reported, and various aspects of the systems have been studied including the relative block length,^20,24,25^ concentration,^26,27^ and solvent.^28,29^ With respect to the core-forming block length, a number of studies have shown that it is possible to determine a relationship between the hydrophobic block DP and the diameter of the nano-object core (*D*_core_).^21,30,31^ Typically, *D*_core_ increases according to a scaling relationship *D*_core_ = *kN*^α^ where *N* is the hydrophobic block length, *k* is a constant related to the Flory–Huggins interaction parameter, and the exponent α provides information on the conformation of the core polymer chains.^23,31−33^ Typically, it is widely accepted that an α value of 0.5 indicates a weakly segregated regime with collapsed hydrophobic chains; while a value of 0.8 indicates a strongly segregated regime comprising stretched core chains with more discreet hydrophobic and hydrophilic domains.^34,35^

Small angle X-ray scattering (SAXS) has become a common method for the characterization of PISA nanoparticles because of the depth of structural information that can be obtained in a non-destructive manner.^25,27,31,32,36,37^ Another major advantage of SAXS is its ability to monitor nanoparticle formation in situ, which has facilitated the ability to gain deep insights into the PISA process. Typically, in situ SAXS requires high photon flux only afforded by synchrotron radiation (SR) X-ray sources due to poor contrast in scattering length density (ξ) between polymers and most solvents. The complexity of these in situ experiments has steadily increased from initial capillary-based systems with temperature-controlled cells to stirred batch reactors.^31,36−38^ However, SR approaches do suffer some limitations, such as limited availability of instrument time, beam damage, or rate acceleration caused by high intensity X-rays.^20^ The latter two problems have been minimized by ensuring that the measured sample is continually exchanged during irradiation using of a custom-stirred cell. However, some rate enhancement was still observed.^36,37^ Herein, we exploit an alternative approach whereby SAXS measurements are conducted on a reaction solution as it exits a continuous-flow reactor operating at steady state (where the reaction time is governed by flow rate). Using the flow cell also enables longer acquisition times, which opens up the opportunity to use lower flux laboratory SAXS instruments, which are both more accessible and less destructive.^39,40^ We use this approach to study the effect of varying the hydrophilic block length on the evolution of spherical nanoparticles during the RAFT aqueous dispersion polymerization of diacetone acrylamide (DAAm).

## Experimental Section

Dimethyl acrylamide (DAAm , 99%), 4,4′-azobis(4-cyanopentanoic acid) (ACVA, 99%), hydrochloric acid (<37%), deuterated methanol (CD_3_OD, 99.9%), and deuterium oxide (D_2_O, 99.9%) were purchased from Sigma-Aldrich. Diacetone acrylamide (DAAm, 99%) was purchased from Alfa Aesar. 2-(Butylthiocarbonothioylthio) propanoic acid (PABTC, 95%) was purchased from Boron Molecular. 2,2-Azobis[2-(2-imidiazolin-2-yl) propane (VA-044, 97%) was purchased from WAKO Chemical. All Reagents were used as received.

### Batch Synthesis of PDMAm_*x*_ Macromolecular Chain Transfer Agent (Macro-CTA)

A typical protocol for a PDMAm_*x*_ macro-CTA was performed as follows. For a target macro-CTA DP of 100, DAAm (35 g, 0.351 mol, 100 equiv), PABTC (0.8408 g, 0.00351 mol, 1 equiv), and ACVA (0.0988 g, 0.351 mmol, 0.1 equiv) were dissolved in dioxane (84 g) in a round-bottom flask containing a magnetic stirrer. This 30% w/w solution was purged with nitrogen for 30 min and followed by immersion in an oil bath set at 80 °C. After 70 min, the polymerization was quenched by exposure to air and subsequently purified by precipitation into a 10-fold excess of diethyl ether. The precipitated yellow product was collected and washed three times with diethyl ether and dried under vacuum for 2 h. ^1^H NMR spectroscopy was used to calculate an average degree of polymerization of 98 by comparing integrals from the two-proton signal at 3.40–3.50 ppm resulting from the RAFT agent with those from the polymer backbone between 2.40 and 2.79 ppm (Figure S1). Good control over the polymerization was maintained as judged by final molar mass dispersity of <1.20, which was the case for all macro-CTAs.

### Flow Reactor Configuration

**Figure 1:**
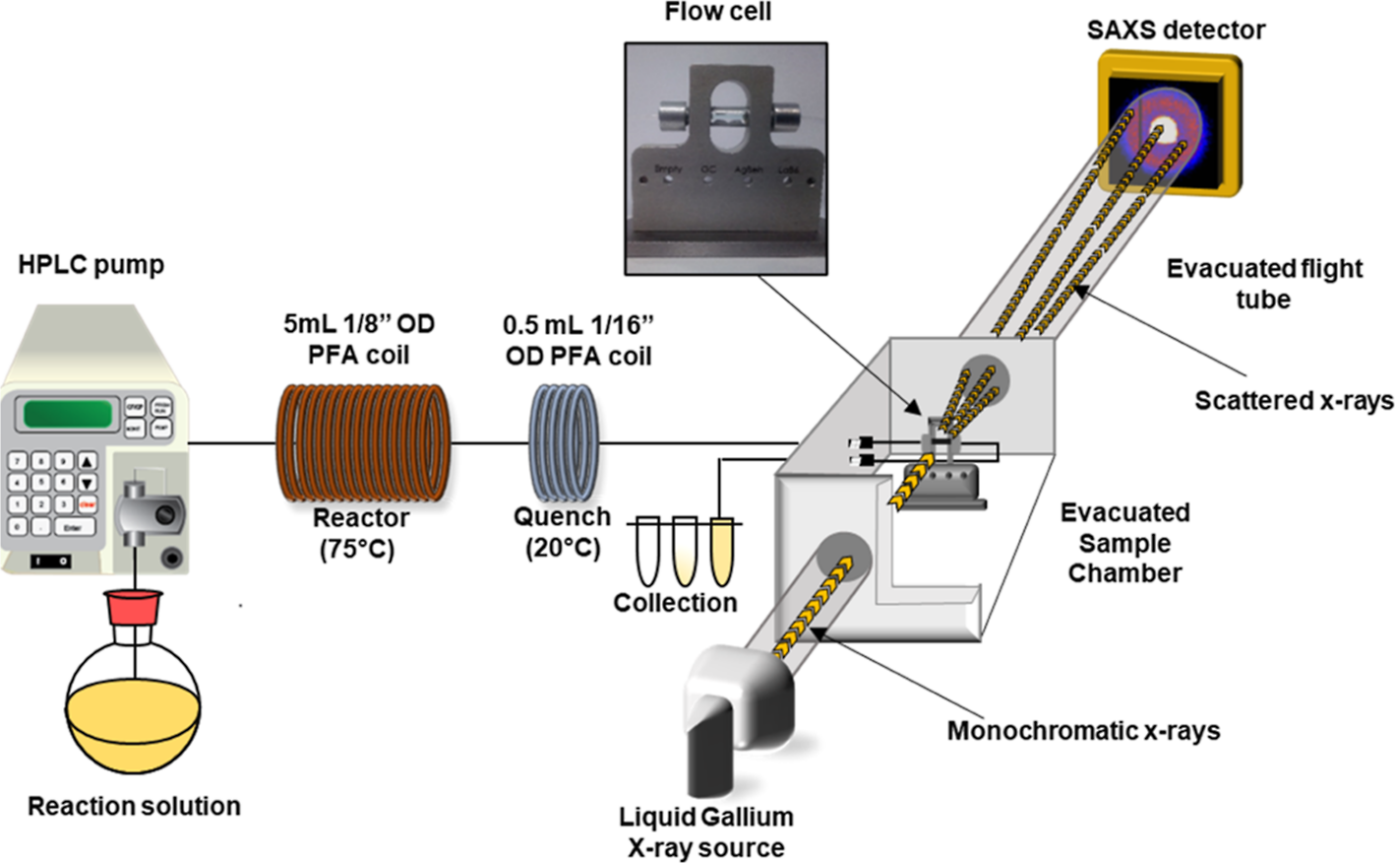
A schematic of the reactor platform comprising a heated tubular coil reactor, a quench coil at 20 °C, a SAXS compatible flow cell in an evacuated sample chamber and sample collection.

The flow-reactor platform (Figure 1) comprised a Jasco PU-1580 HPLC pump connected to a 5 mL perfluoroalkoxy (PFA) (1/8”OD, 1.6 mm ID) reactor coil which was immersed in a water bath at 75 °C. Downstream from the reactor, 30 cm of PFA tubing (1/16” OD, 0.8 mm ID) was immersed in a water bath at ambient temperature which cooled the reaction solution, thus quenching the polymerization. A further section of PFA tubing then routed the solution into the glass flow cell mounted within the SAXS instrument sample chamber. Upon exiting the reactor, the solution was routed through a 100 psi back pressure regulator (BPR) which maintained a constant flow rate and positive pressure which minimized oxygen permeation through the permeable PFA tubing.

### Flow Synthesis of PDMAm_*x*_–PDAAm_400_

A typical procedure for the aqueous synthesis of PDMAm_*x*_–PDAAm_400_ at 10% w/w was as follows: for a target composition of PDMAm_47_–PDAAm_400_, PDMAm_47_ macro-CTA (0.390 g, 0.072 mmol, 1 equiv), DAAm (5 g, 0.03 mol, 400 equiv), 2,2′-azobis(2-(2-imidazolin-2-yl)propane) dihydrochloride (VA-044; 0.00484 g, 0.014 mmol, 0.2 equiv) were dissolved in pH 2.5 deionized water (48.47 g) in a 250 mL round-bottomed flask. The reaction solution was then pumped through the experimental setup outlined above at flow rates of 5, 3, 1, 0.8, 0.6, 0.4, and 0.2 mL min^–1^ to generate residence times of 60, 100, 300, 380, 500, 750, and 1500 s (calculated by eq 1) with monomer conversion exceeding 94% achieved after 1500 s. The residence time is calculated as
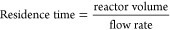
1

The flow rate on the pump was adjusted to achieve the desired residence time within the reactor coil. While the flow rate could be altered to generate almost any reasonable residence time in theory, from previous batch reactions it was found that high conversion would be achieved within 15 min, as such a maximum residence time of 1500 s was selected. Each experiment was deemed to reach steady state after passing three reactor volumes of reaction material (15 mL), which is typical for experiments of this nature,^41^ at which point collection of material and SAXS analysis was performed. A continuous flow methodology was used, while transient methods were considered due to the 5 min acquisition time required per scan, this was not possible using laboratory SAXS sources.

### Transient Kinetic Studies

Transient kinetic studies (a.k.a time-sweeps) were performed using the flow reactor configuration above, but with the outlet proceeding through a Magritek Spinsolve Ultra 60 MHz benchtop NMR instrument instead of a SAXS flow cell. Once the coil reactor was equilibrated to the desired temperature, the pumps were set at 5 mL min^–1^ to fully load the coil. Once at steady state, the flow rate was reduced to 1 mL min^–1^ for 20 minutes whilst continuously recording ^1^H NMR spectra with each measurement comprising two scans, each with an acquisition time of 6.4 s were continuously taken. The flow rate was subsequently reduced to 0.2 mL min^–1^ and the process of acquisition was repeated. Determination of conversion was calculated by comparing the integrals of the vinyl protons between 5.50 and 6.50 ppm relative to those obtained value for a sample obtained for the initial reaction solution (considered to be *t*_0_), this is possible due to no change in concentration occurring.

### Small-Angle X-ray Scattering

SAXS patterns were recorded using a Xeuss 3.0 laboratory SAXS instrument (located at Diamond Light Source, UK). A monochromatic X-ray beam was generated from a gallium alloy Metaljet source (9.2 keV) and collimated to generate a beam of 0.4 mm in diameter. Scattering patterns were obtained with an acquisition time of 5 min with a *q* range of 0.0023–0.71 Å^–1^ using an Eiger 1 M detector, where  with θ being half of the scattering angle. All scattering patterns were processed using standard methods including background subtraction of the solvent, beam, and vessel and 2D image integrated to generate a 1D plot of intensity *I*(*q*) vs *q* using the diamond software *DAWN* .^42,43^ The SAXS patterns were fit to a spherical micelle model combined with a structure factor based upon the corresponding Percus–Yevick hard sphere model.^44^ This approach is analogous to that taken by Derry et al.,^31^ who describe it in detail including the adaption of the original Spherical micelle model reported by Pedersen.^45,46^ The individual scattering patterns were fitted using the Irena fitting Package within on Igor Pro v8.^47,48^ The *q*-range studied enabled determination of particle core radius (*R*_c_), volume fraction of solvent and unreacted monomer within the core (*x*_sol_), and volume fraction of interacting nanoparticles within the system (volume fraction). It should be noted that the hard sphere structure factor is non-ideal for such polymer nanoparticles and therefore provides only qualitative information. All scattering patterns were obtained at reaction concentration of 10% w/w.

### High Field ^1^H NMR Spectroscopy

High resolution ^1^H NMR spectra on the final products were recorded using either a 400 or 500 MHz Bruker NMR spectrometer. All samples were dissolved in D_2_O, CDCl_3,_ or CD_3_OD. Chemical shifts are reported in ppm. The average number of scans accumulated per spectrum was typically 64. Monomer conversion was determined by monitoring the integrals of the vinyl signals at 5.5–6.5 ppm relative to the signals resulting from the pendant methyl protons from the DAAm monomer and PDAAm polymer between 2.1 and 2.4 ppm (Figure S2).

### Gel Permeation Chromatography (GPC)

GPC studies were conducted using an Agilent 1260 infinity system equipped with two 300 mm Mixed-C columns plus a 5 μm guard column and refractive index detector. Dimethylformamide with 0.1% w/v lithium bromide was used as eluent. Both the detector and the column over were heated to 60 °C. A series of nine near-monodisperse poly(methyl methacrylate) calibration standards (*M*_p_ ranging from 885 to 2 200 000 g mol^–1^) in conjunction with the RI detector to determine molecular weights and molecular weight dispersity.

### Dynamic Light Scattering (DLS)

DLS was conducted at 25 °C using Malvern instruments Zetasizer Nano with all copolymer dispersions diluted to 0.1% w/w prior to measurement. Back-scattered light was detected at 173°. Intensity average hydrodynamic diameter was determined via the Stokes–Einstein equation assuming spherical and independent particles using a non-negative least squares algorithm. The mean *z*-average particle diameter and DLS polydispersity index (PDI) were averaged over three consecutive runs with a minimum of five runs per measurement.

## Results and Discussion

### Synthesis of PDMAm Macro-CTA

**Figure 2:**
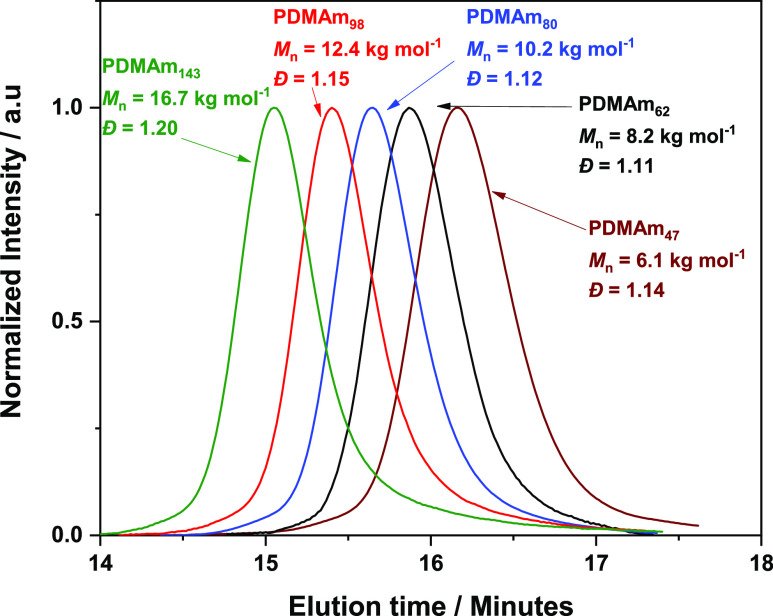
GPC chromatograms of a series of PDMAm_*x*_ macro-CTAs with DPs ranging from 47 to 143, demonstrating the systematic shift in *M*_n_ with increasing PDMAm DP.

**Scheme 1:**
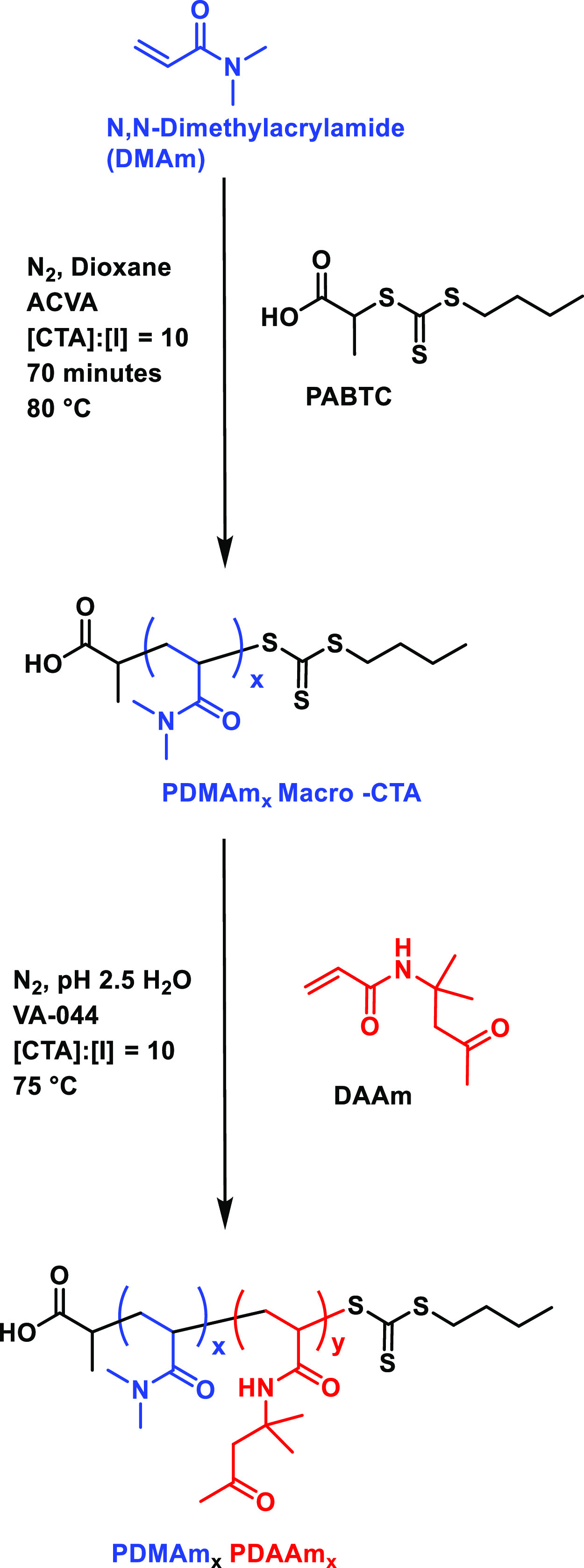
Two-step synthesis of PDMAm_*x*_–PDAAm_400_: initial synthesis of PDMAm_*x*_ macro-CTA via RAFT solution polymerization of dimethylacrylamide in the presence of PABTC using ACVA as the thermal initiator at 30% w/w in dioxane: This was subsequently precipitated by addition to excess diethyl ether. The PDMAm macro-CTA was subsequently chain extended with DAAm via aqueous RAFT dispersion polymerization using VA-044 as the initiator.

A series of five PDMAm_*x*_ macromolecular chain transfer agents (macro-CTAs) with varying degrees of polymerization (DP) were synthesized via RAFT polymerization in dioxane using a standard batch procedure outlined above (Scheme 1).^24^ After purification, a series of PDMAm_*x*_ macro-CTAs were obtained where *x* = 47, 62, 80, 98, and 143 as judged by high field ^1^H NMR end group analysis. GPC confirmed a monomodal molecular weight distribution and a monotonic increase in molecular weight with degree of polymerization (Figure 2). Low molar mass dispersitys (*D̵* < 1.20) were achieved in all cases indicating well-defined macro-CTAs.

An appropriate residence time range for the subsequent flow-SAXS studies was determined using the transient kinetic study at 75 °C, where online NMR studies (Figure S3) indicated >90% conversion was achieved after a residence time of approximately 1200 s. To avoid possible artefacts caused by conducting wide flow rate sweeps,^49^ the conversion determined at short residence times was not used due to the flow rate greater than 1 mL min^–1^ resulting in high signal to noise in the obtained NMR spectra.

Using these conditions, a series of in situ SAXS flow studies were conducted on PDMAm_*x*_–PDAAm_400_ copolymer synthesis via RAFT aqueous dispersion polymerization where *x* was varied between 47 and 143. Conversion values obtained from measurements calculated upon collection of the sample from the outlet at steady state were in good agreement with the transient measurements (Figure S3). Steady-state conversion measurements obtained for all samples indicated slow polymerization up until around 300 s followed by a large increase indicative of a rate enhancement as expected for PISA following self-assembly.^50^ Unfortunately, the exact nature of this could not be discerned due to only a few samples being obtained at short residence times (since this would be extremely material intensive).

**Figure 3:**
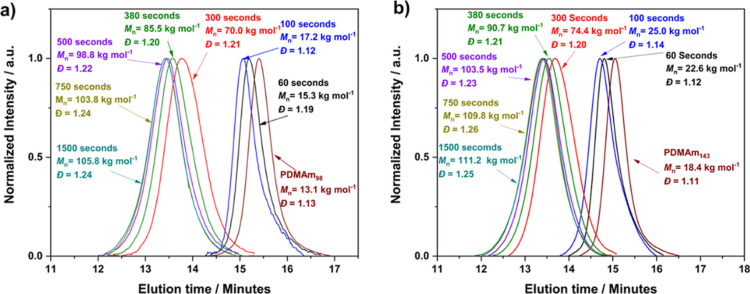
GPC chromatograms during RAFT polymerization targeting (a) PDMAm_98_–PDAAm_400_ and (b) PDMAm_143_–PDAAm_400_ including PDMAm_*x*_ macro-CTA and at residence times varying between 60 and 1500 s. A systematic shift to the lower elution times as residence time increased indicate a growth in PDAAm block length until 750 s after which there is negligible growth with increased residence time.

Chromatograms obtained by GPC for the reactions all showed a systematic shift in the peaks to higher molecular weight distributions, confirming successful chain extension. For example, when targeting PDMAm_98_–PDAAm_400_*M*_n_ values increased from 70.0 to 105.8 kg mol^–1^ between residence times of 60 to 1500 s. In all cases, the molar mass dispersity remained below 1.30, which is in a similar range to that observed by Parkinson et al. for a similar PFA reactor.^50^ The sample obtained for 1500 s was similar to that at 750 s indicating no further polymerization (and hence particle growth) beyond this point, which corroborated with the kinetics studies (Figures S3 and S4). A particularly large shift in molecular weight, from 17.2 to 70.0 kg mol^–1,^ was observed between 100 and 300 s, which can be explained by the point of nucleation and resulting rate acceleration occurring within this range (Figure 3a).^51^ However, the apparent turbidity in the samples obtained at 100 s indicated the presence of nucleated particles before rate enhancement. Similar observations were made by Takahashi et al. where rate enhancement was not directly correlated with the onset of turbidity.^52^ The observations for the remaining macro-CTAs were similar in all cases with a marked increase in *M*_n_ observed between 100 and 300 s followed by sequential increase in *M*_n_ with residence time (Figures 3b and S5–S7). For all reactions, a linear increase in *M*_n_ vs conversion indicated a well-controlled RAFT polymerization (Figure S8).

**Figure 4:**
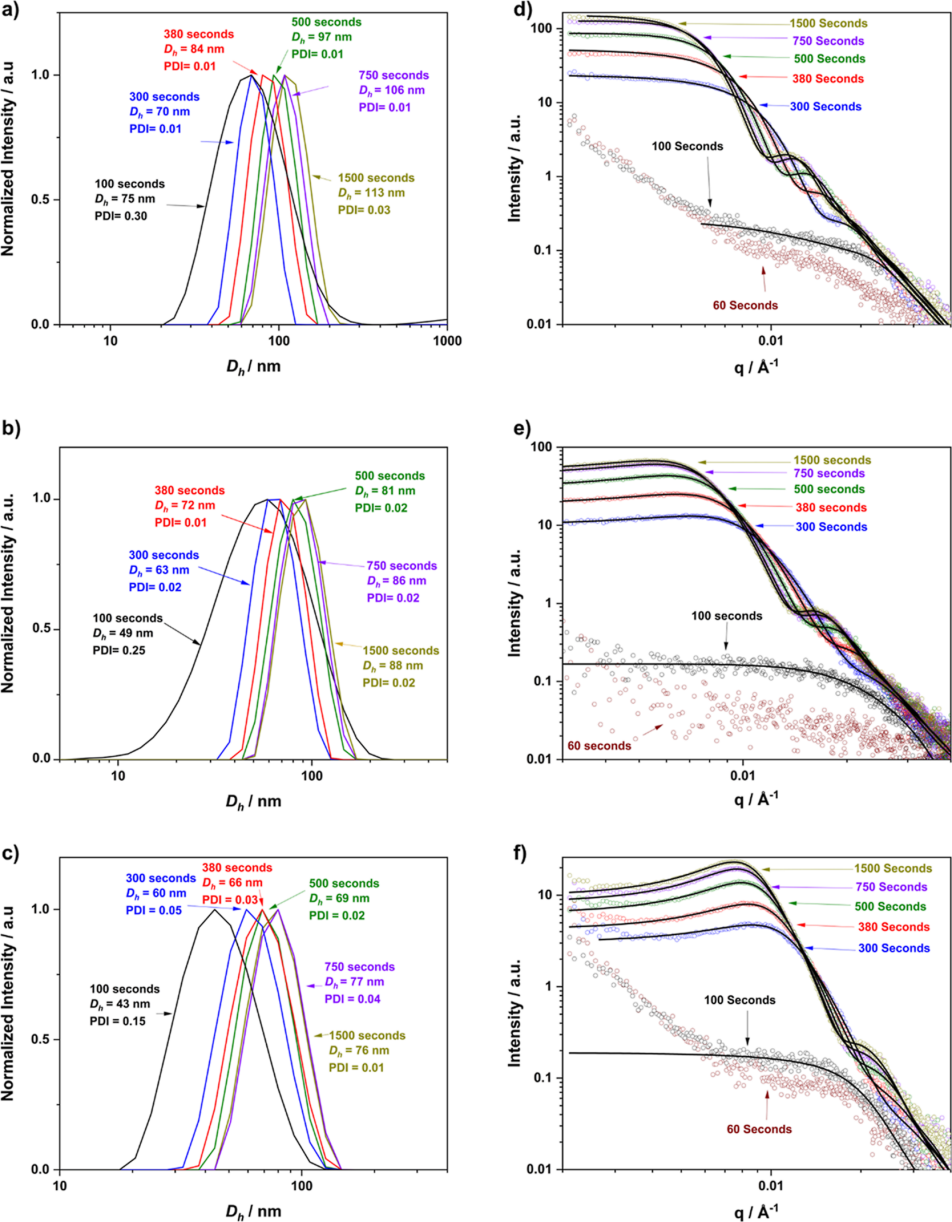
(a–c) Offline DLS traces obtained for samples collected at various residence times demonstrating particle growth during the synthesis of PDMAm_47_–PDAAm_400_, PDMAm_98_–PDAAm_400,_ and PDMAm_143_–PDAAm_400._ (d–f) In situ SAXS patterns obtained for the same reactions where experimental SAXS data are shown as hollow circles whilst the fitted model is indicated by the solid line. Particles were found to have formed at 100 s in all cases but were highly disperse compared to higher residence times (typical DLS PDI 0.25–0.37 compared to 0.01–0.02 thereafter). Intensity has been normalized to aid in comparison.

Dynamic light scattering (DLS) studies conducted on the reactions confirmed the presence of nanoparticles in all cases apart from after 60 s (Figures 4a–c, S9 and S10). It is particularly notable that all the 100 s samples show a broader size distribution and associated larger PDI, between 0.15 and 0.38 compared to later samples, where narrow size distributions with PDIs <0.05 were obtained for particle synthesized using all macro-CTAs. TEM analysis of PDMAm_98_ confirmed the formation of spherical nanoparticles (Figure S11).

The online SAXS measurements provided significantly more detailed insights into the PISA process (Figures 4d–f and S12). Qualitative analysis of the SAXS data indicated similar behavior for each macro-CTA where weak scattering was obtained after 100 s, indicating a population of weakly scattering objects likely loosely associated chains. As the reaction proceeded, the relative overall intensity of the patterns indicated an increasing volume of objects as would be expected during the PISA process.^36,53^ Between 60 and 100 s, this increase is relatively small, presumably due to the fact that the majority of the material is in a solvated state. Fitting the data to a spherical core shell model combined with a hard sphere structure factor^54,55^ indicated a relatively high volume fraction of solvent and unreacted monomer in the core (*x*_sol_ > 0.8; Table 1) in all cases confirming the presence of relatively large, loosely aggregated objects.

**Table 1:** Summary of Ex Situ GPC and DLS and In Situ SAXS Data obtained at different time points for the copolymers synthesized in this work: GPC was used to obtain the number average molecular weight (*M*_n_) and molar mass dispersity (*D̵*)^a^

PDMAm_*x*_ DP	residence time (s)	*M*_n_ (g mol^–1^)	*D̵*	*D*_h_ (nm)	PDI	*N*_agg_	*x*_sol_	*R*_core_ (nm)	*R*_g,_ (nm)	*D*_particle_ (nm)	*R*_structure_ (nm)	volume fraction
		GPC		DLS		SAXS						
47	0 (macro-CTA)	6300	1.11									
	60	10 000	1.11									
	100	12 000	1.10	75	0.30	13	0.82	4.5	2.4	17.6	9.9	0.19
	300	46 700	1.18	70	0.01	1420	0.32	30.2	2.2	69.1	27.8	0.10
	375	61 100	1.19	84	0.01	2018	0.23	36.1	2.2	80.9	32.7	0.12
	500	74 000	1.22	97	0.01	2581	0.15	40.8	1.5	87.8	41.3	0.12
	750	81 700	1.24	106	0.01	3426	0.06	45.1	1.5	96.3	46.6	0.13
	1500	84 000	1.23	113	0.03	3906	0.04	47.2	1.9	102.2	45.7	0.12
62	0 (macro-CTA)	8200	1.11									
	60	11 900	1.10									
	100	12 700	1.20	48	0.37	29	0.94	9	1.4	23.4	11.2	0.05
	300	59 800	1.21	73	0.02	833	0.25	26.9	2.3	63.0	33.5	0.11
	375	67 800	1.30	82	0.02	1200	0.18	31	2.2	71.1	37.7	0.12
	500	77 600	1.28	90	0.02	1477	0.13	34.6	2.1	77.9	40.8	0.13
	750	83 000	1.28	96	0.02	1904	0.05	37.1	1.9	82.1	42.7	0.13
	1500	82 300	1.28	102	0.05	2142	0.03	38.3	2.2	85.6	42.7	0.11
80	0 (macro-CTA)	10 200	1.12									
	60	13 900	1.10									
	100	16 800	1.06	57	0.38	26	0.65	6.7	1.5	19.6	12.4	0.08
	300	64 900	1.20	66	0.02	580	0.14	23.3	2.9	58.2	30.3	0.11
	375	77 800	1.20	75	0.02	799	0.1	27.3	2.2	63.2	35.2	0.13
	500	95 000	1.21	85	0.02	1044	0.06	31.8	2.0	71.7	39.8	0.15
	750	101 300	1.22	90	0.02	1364	0.03	35.3	2.1	79.0	42.9	0.16
	1500	100 000	1.27	93	0.05	1476	0.02	36.3	2.1	81.2	43.7	0.16
98	0 (macro-CTA)	13 100	1.13									
	60	15 300	1.19									
	100	17 200	1.12	43	0.15	12	0.84	7.6	1.2	20.1	17.6	0.05
	300	70 000	1.21	63	0.03	173	0.56	21	2.9	53.6	29	0.14
	375	85 500	1.20	72	0.01	307	0.4	24.7	3.0	61.1	31.3	0.15
	500	98 800	1.22	81	0.02	443	0.3	27.8	2.7	66.3	36.1	0.16
	750	103 800	1.24	86	0.02	856	0.12	30.6	2.8	72.5	37.2	0.16
	1500	105 800	1.24	88	0.01	918	0.07	31.6	2.7	74	38.9	0.16
143	0 (macro-CTA)	18 400	1.11									
	60	22 600	1.12									
	100	25 000	1.14	43	0.15	107	0.84	11.5	3.8	38.5	13.9	0.14
	300	74 400	1.20	60	0.05	164	0.47	18	2.5	45.9	26.8	0.16
	375	90 700	1.21	66	0.03	225	0.32	20.1	2.6	50.8	29.6	0.19
	500	103 500	1.23	69	0.02	264	0.31	22.4	2.5	54.8	32.4	0.21
	750	109 800	1.26	77	0.04	403	0.08	23.9	2.6	58.4	34.1	0.22
	1500	111 200	1.25	76	0.01	466	0.02	24.7	2.5	59.3	34.9	0.22

a DLS was used to obtain the *z*-average hydrodynamic diameter (*D*_h_) and polydispersity index (PDI). Flow-SAXS enabled determination of aggregation number (*N*_agg_), volume fraction of solvent and unreacted monomer within the core (*x*_sol_), radius of the particle core (*R*_c_), radius of the gyration of the individual corona chains (*R*_g_), structure factor radius (*R*_structure_), and volume fraction of interacting nanoparticles in the system based on the Percus–Yevick hard sphere model. The overall particle size (*D*_particle_) is calculated from the SAXS data where: *D*_particle_ = 4*R*_g_ + 2*R*_core_.

Further progression in the polymerization resulted in a decrease in *x*_sol_ indicating that solvent is expelled, initially coinciding with a marked increase in scattering intensity between 100 and 300 s^38^ This suggests a transition from loosely aggregated low molecular weight chains to more defined, narrower PDI micelles (also suggested by DLS) comprising of higher MW copolymer chains (as recorded by GPC). The consolidation of these observations provides a direct observation of a pre-aggregation step analogous to pre-nucleation during the two-step crystallization process.^56^ It is likely that these pre-aggregates do not provide a sufficiently hydrophobic environment to cause partitioning of the monomer into the core, which would result in a rate acceleration. Although no kinetic data was obtained, it is indeed implied by a large increase in *M*_n_ observed by GPC after 100 s. A similar identification has been previously observed during in situ SAXS monitoring of aqueous emulsion polymerization of 2,2,2-trifluroethyl methacrylate by Czajka and Armes as a structure was determined to be formed by the increase in *I*(*q*) by SAXS prior to that determined via DLS.^36^

SAXS patterns recorded for polymerizations using macro-CTA DPs ranging from 47 to 98 had a zero gradient at low *q* once aggregation had occurred (after 300 s), which is indicative of the presence of spherical nanoparticles. However, it should be emphasized that the relatively high concentration (10% w/w) of these measurements means the patterns have a structure factor, which can complicate analysis in this region. This becomes gradually more prominent at longer PDMAm DPs, being particularly evident in the longest PDMAm_143_ macro-CTA, manifesting itself as a peak at approximately 0.01 Å^–1^, which moves to lower *q* as the polymerization progresses. This corresponds to an increasing length scale which contradicts the expectation that larger particles should approach more closely. Instead, it may indicate that the corona chains provide better steric stabilization as the particles grow. Given the stabilizer length does not change during the polymerization, monomer plasticization may facilitate closer interaction at lower conversion. This is further supported by the SAXS-determined volume fraction of interacting nanoparticles within the system increasing with PDMAm DP for samples after 1500 s (Figure S13). The fact that this observation is enhanced for the longer macro-CTA may give insights into the hypothesis that their enhanced steric stabilization prevents cooperative collisions that typically enable the order–order morphological transition (e.g., worms or vesicles).^51^

**Figure 5:**
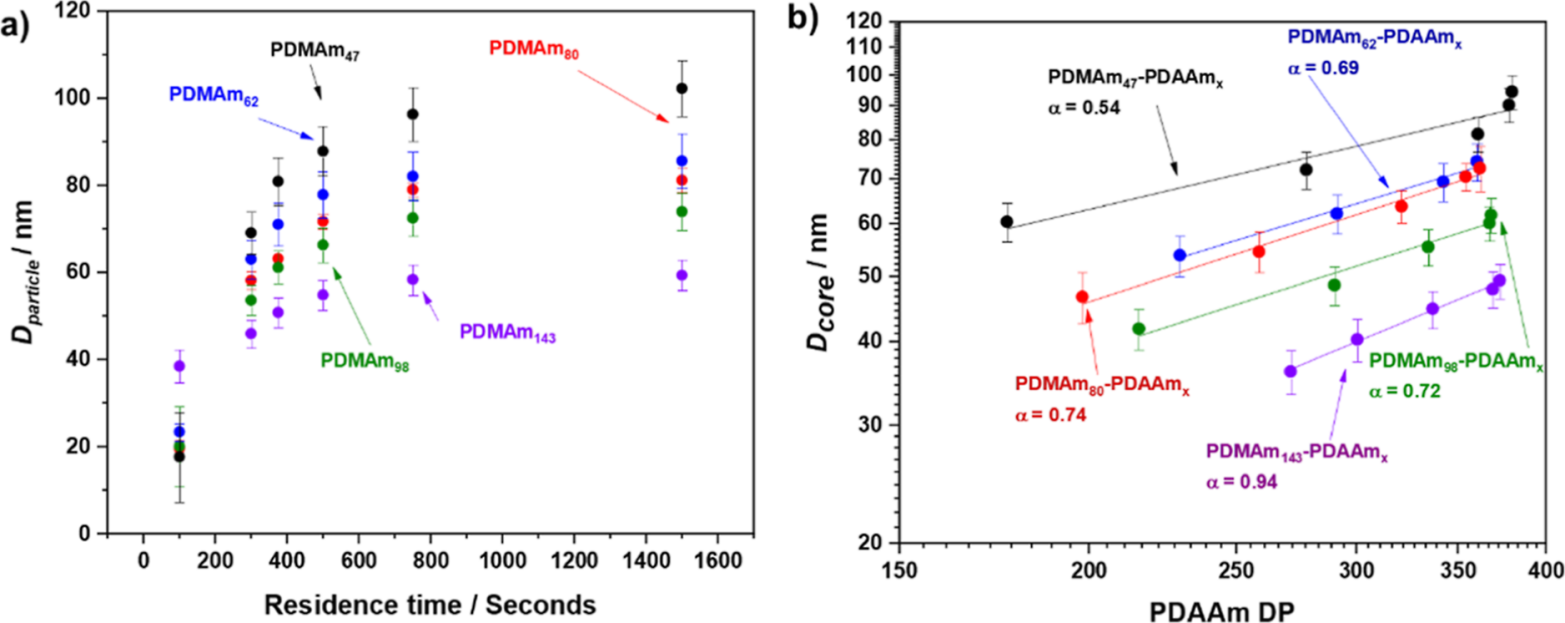
(a) Diameter of the particle (*D*_Particle_) as a function of residence time for a series of PDMAm_*x*_–PDAAm_400_ copolymers synthesized by RAFT dispersion polymerization were *x* = 47, 62, 80, 98, and 143. (b) *D*_core_ with PDAAm DP when targeting a PDAAm DP of 400 at different macro-CTA block lengths. Error bars indicate the standard deviation of the *D*_core_.

Increasing the residence time beyond 300 s gives the characteristic growth of spherical PISA nanoparticles with the shift of the form factor peak around 0.030 to 0.015 Å^–1^ indicating a growth in particle size with residence time. Fitting this feature enabled determination of *D*_core_ as a function of time, with significant particle growth between 100 and 750 s (Figure 5a).

It was observed that *D*_core_ was inversely related to PDMAm_*x*_ DP despite the core DP being similar, with the core size for the PDMAm_143_–PDAAm_400_ spheres being approximately half that of the PDMAm_47_–PDAAm_400_ spheres. This correlates with previous studies by Akpinar et al. in which a similar decrease in size was observed with increase of the hydrophilic block length of poly(glycerol monomethacryalate) synthesized by aqueous emulsion polymerization.^25^ By calculating the *N*_agg_ from these values, it can be seen that there are significantly fewer copolymer chains per particle as the macro-CTA DP is increased (Table 1).^57^ This is a reasonable conclusion given that the longer hydrophilic block will impart a higher curvature at the interface, meaning fewer chains can pack in the particle and has been reported on numerous occasions in the literature.^25,38,57^ This will also affect the conformation of the chains in the core, which can be elucidated from the relationship between core DP and core radius according to *D*_core_ = *kN*^α^ where the exponent, α, provides information on the conformation of the solvophobic block within the nano-object and *k* is a constant derived from the Flory–Huggins interaction parameter.^21,33^ For the three intermediate macro-CTA DPs, an exponent α between 0.69 and 0.74 suggests that the copolymer chains are in a strongly segregated regime often observed for such self-assembled systems, which typically range between 0.5 and 0.8 (Figure 5b).^34,37,57^ For the extreme macro-CTA DPs of 47 and 143, markedly different α values of 0.47 and 0.94 are observed suggesting a different copolymer chain arrangement in the form of weakly segregated regime and a super-strong segregation regime, respectively,^34,35^ which is potentially complicated by the partitioning of monomer and solvent within the cores. It should also be noted that values obtained from samples with core PDAAm DPs below 50 were removed from fitting due to the clearly different morphology and high *x*_sol_.

**Figure 6:**
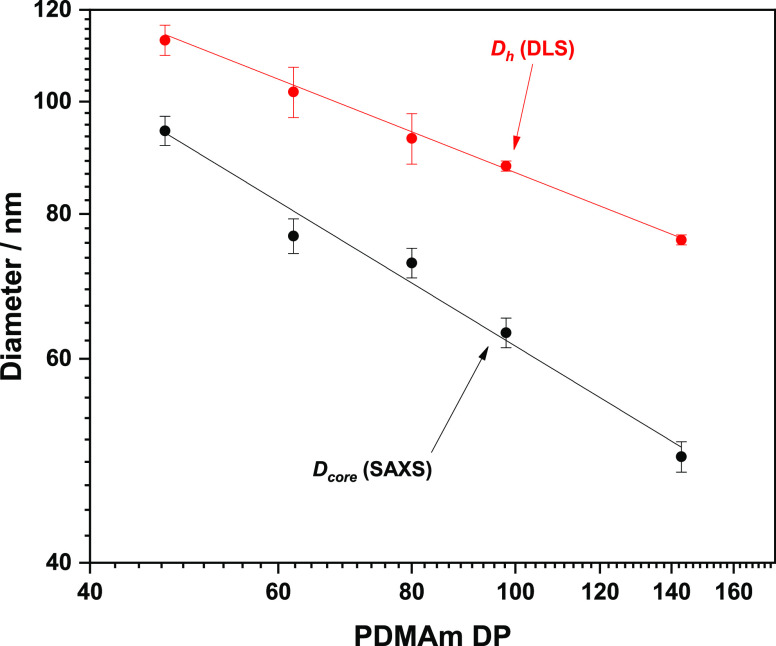
Hydrodynamic diameter (*D*_h_; determined by DLS) and core diameter (*D*_core_; determined by SAXS) as a function of PDMAm degree of polymerization for samples obtained after 1500 s. Error bars shown are determined by the determined PDI of the hydrodynamic diameter.

**Figure 7:**
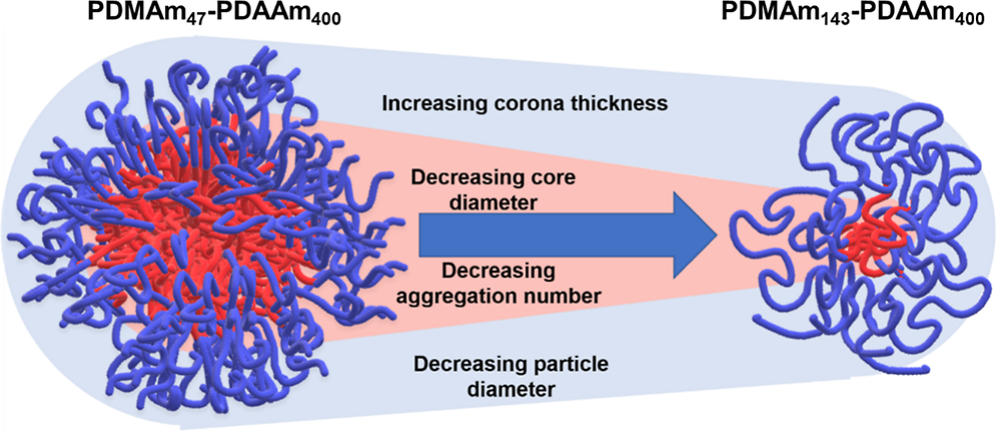
Schematic representation of the change in particle dimensions when the DP of the PDMAm macro-CTA is increased from 47 to 143 while maintaining a constant PDAAm core DP of approximately 400.

Finally, the high-conversion samples’ *D*_h_ (measured by DLS) was compared to *D*_core_ (measured by SAXS) to determine whether any insights into how the corona contributes to the overall size of the particle (Figure 6). The trend of decreasing diameters with increasing PDMAm DP was in agreement for both DLS, but the difference between *D*_h_ and *D*_core_ increased as a function of increasing PDMAm DP. This provides a crude indication that the area of solvation around the polymer nanoparticles also increases (Figure 7) attributed by the ability of the higher molecular weight PDMAm chains to hold on to more water molecules.

## Conclusions

A flow-reactor equipped with an X-ray transparent flow cell at the outlet has facilitated the use of a low-flux laboratory SAXS instrument for in situ monitoring of the formation and morphological evolution of PDMAm_*x*_–PDAAm_400_ spherical nanoparticles during RAFT aqueous dispersion polymerization (where *x* = 47–143). For all reactions, GPC and NMR indicated that the polymerization proceeded as expected. Qualitative analysis of the in situ SAXS data indicated a pre-micellization stage at 100 s as judged by the absence of a form factor peak. Upon fitting the data to a spherical micelle model, the presence of diffuse nanoparticles with highly solvated cores was observed. A polydisperse size distribution obtained by DLS supported this observation. Between 100 and 300 s, a form factor peak becomes prominent in the SAXS patterns coinciding with a considerable reduction in the volume of solvent in the particle cores. Further progression of the polymerization results in a gradually increasing core diameter which results from both the growing copolymer chains, and an increase in aggregation number facilitated by co-operative collisions between particles. For PISA syntheses conducted using different macro-CTA DPs, *R*_core_ decreased from 47 to 25 nm despite a constant PDAAm core forming target DP of 400. The evolution of *R*_core_ during each reaction also indicated a change in copolymer chain conformation by fitting of each set of data to a power law relationship. As PDMAm DP increases from 47 to 143, the exponent increases from 0.47 to 0.94 suggesting a change from weakly segregated (collapsed chains) regime and a super-strong segregation regime (stretched chains). The intermediate PDMAm DP samples had exponents from 0.69 to 0.74, which is well within the regime expected for this class of particles. Finally, combining observations from both DLS and SAXS, it is apparent that despite the reducing overall diameter of the particles, the apparent corona thickness itself increases.

## Data Availability

The data from this manuscript is available at https://doi.org/10.5518/1328.

## Supporting Information Available

^1^H NMR spectra for the RAFT agent, macro-CTA PDMAm and PDMAm–PDAAm block copolymer. NMR data obtained from steady state and transient kinetic studies targeting PDMA_98_–PDAAm_400_, additional annotated GPC chromatograms, SAXS scattering patterns, and DLS size distributions obtained for samples obtained during in situ studies not detailed in the main manuscript; plot indicating the correlation of volume fraction of nanoparticles within the system as a function of PDMAm DP (PDF)
