# Correction to “Continuous-Flow Laboratory SAXS for In Situ Determination of the Impact of Hydrophilic Block Length on Spherical Nano-Object Formation during Polymerization-Induced Self-Assembly”

**DOI:** 10.1021/acs.macromol.3c01712

**Published:** 2023-09-08

**Authors:** Jonathan D. Guild, Stephen T. Knox, Sam B. Burholt, Eleanor M. Hilton, Nicholas J. Terrill, Sven L. M. Schroeder, Nicholas J. Warren

Reference 17 is not correct and should be as follows:

(17) BlanazsA.; RyanA. J.; ArmesS. P.Predictive phase diagrams for RAFT aqueous dispersion polymerization: Effect of block copolymer composition, molecular weight, and copolymer concentration.Macromolecules2012, 45, 5099−5107.10.1021/ma301059r

