# Supplementary material for: Continuous-Flow Laboratory SAXS for In Situ Determination of the Impact of Hydrophilic Block Length on Spherical Nano-Object Formation during Polymerization-Induced Self-Assembly
Source: Macromolecules. 2023 Aug 4;56(16):6426–35. doi: 10.1021/acs.macromol.3c00585 (PMC10448749; doi:10.1021/acs.macromol.3c00585)
Supplement: Supplementary file 1 — ma3c00585_si_001.pdf [file ma3c00585_si_001.pdf]

Supporting information for:

Continuous-flow Laboratory SAXS for *in situ* Determination of the Impact of Hydrophilic Block Length on Spherical Nano-object Formation during Polymerisation Induced Self-assembly

Jonathan D. Guild, Stephen T. Knox, Sam B. Burholt, Eleanor. M Hilton, Nicholas J. Terrill,

Sven L.M Schroeder and Nicholas J. Warren

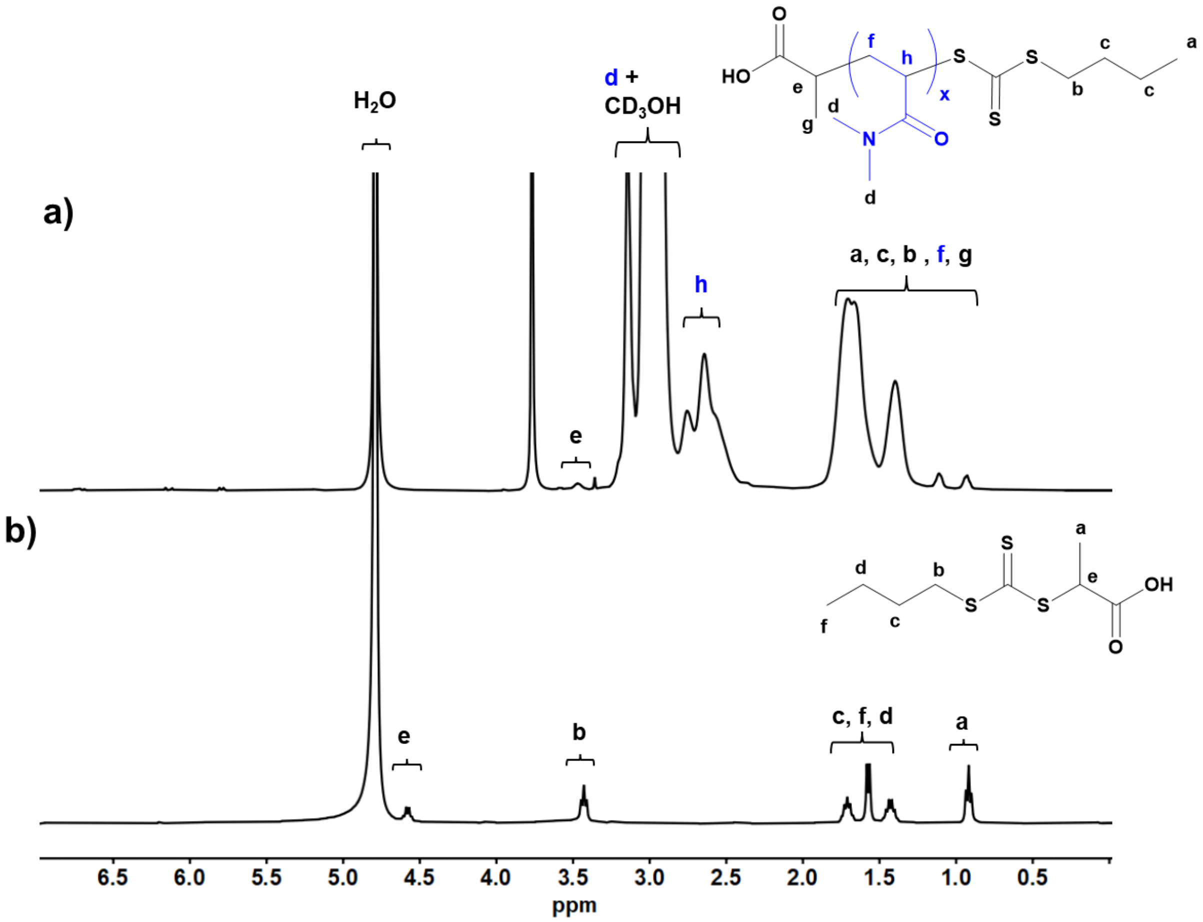

Figure S 1 . ^{1}H NMR spectra for **a)** PABTC RAFT chain transfer agent used within this study, **b)** a $PDMA_{98}$ macro-CTA with intensity sufficient to show key RAFT agent peak (b) which was used to determine PDMAm DP by comparison to backbone peak h.

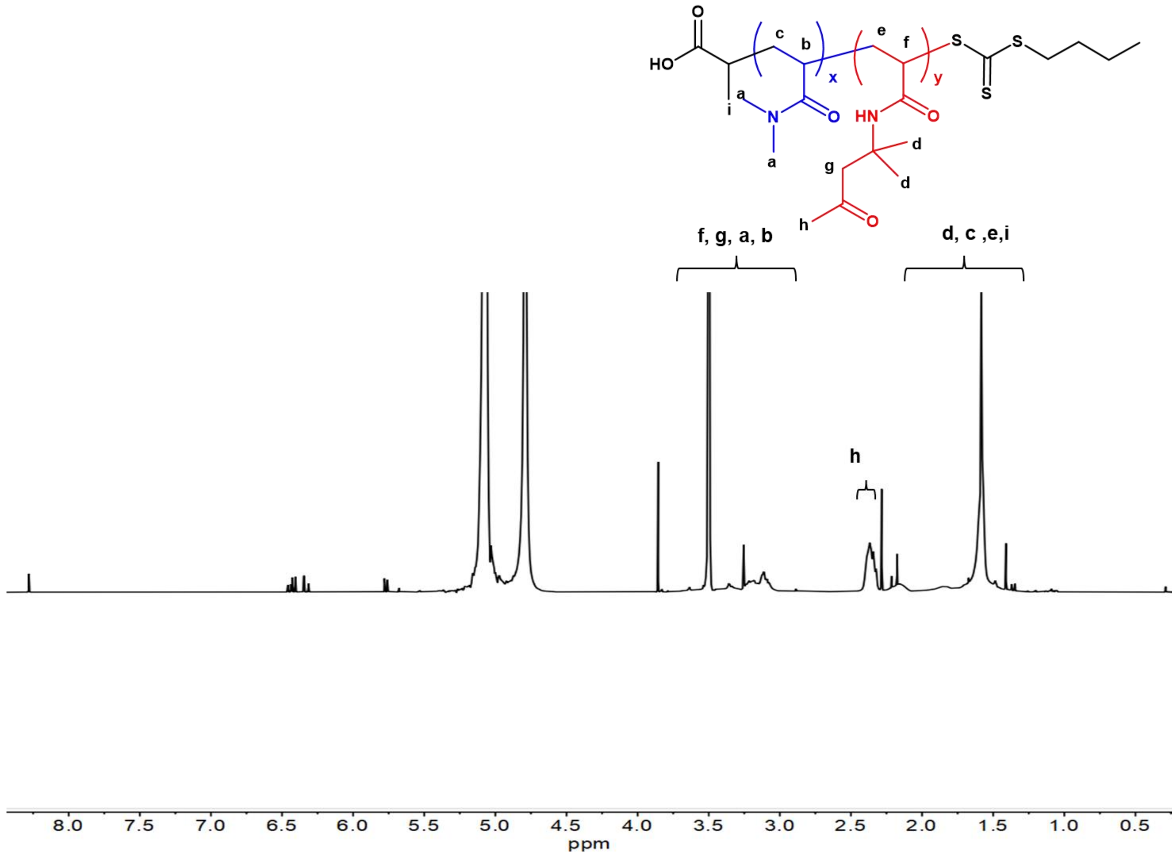

Figure S 2. . ^{1}H NMR spectra recorded for a sample obtained at a residence time of 750 s for a target composition of $PDMAm_{98}$-$PDAAm_{400}$ (sample dissolved in CD_3OD).

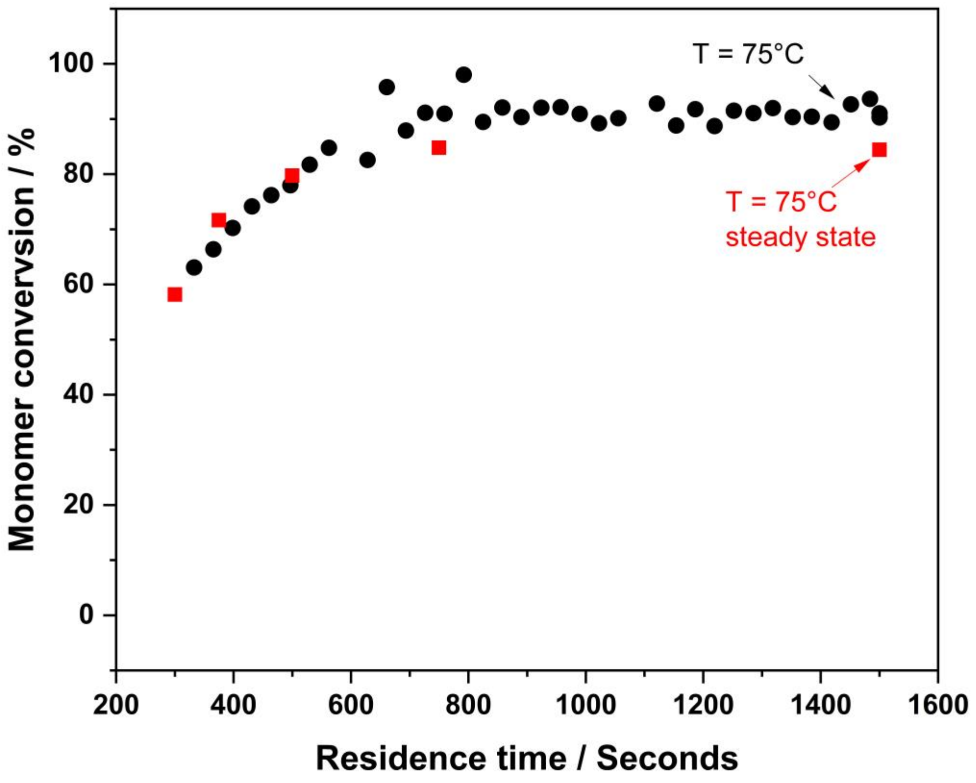

Figure S 3. Transient kinetic profile obtained for the RAFT aqueous dispersion polymerisation of diacetone acrylamide in the presence of a $PDMAm_{98}$ macro-CTA at 75 °C. The study was conducted at 10 % w/w solid. The flowrate was changed from 1 mL min^{-1} to 0.2 mL min^{-1} and NMR spectra were obtained continuously with a 6.4 s acquisition time at 2 scans. A maximum monomer conversion obtained at approximately1500 seconds was 92 %. Steady state measurements were obtained following three reactor volumes passing at the corresponding flow rate to generate the given residence time.

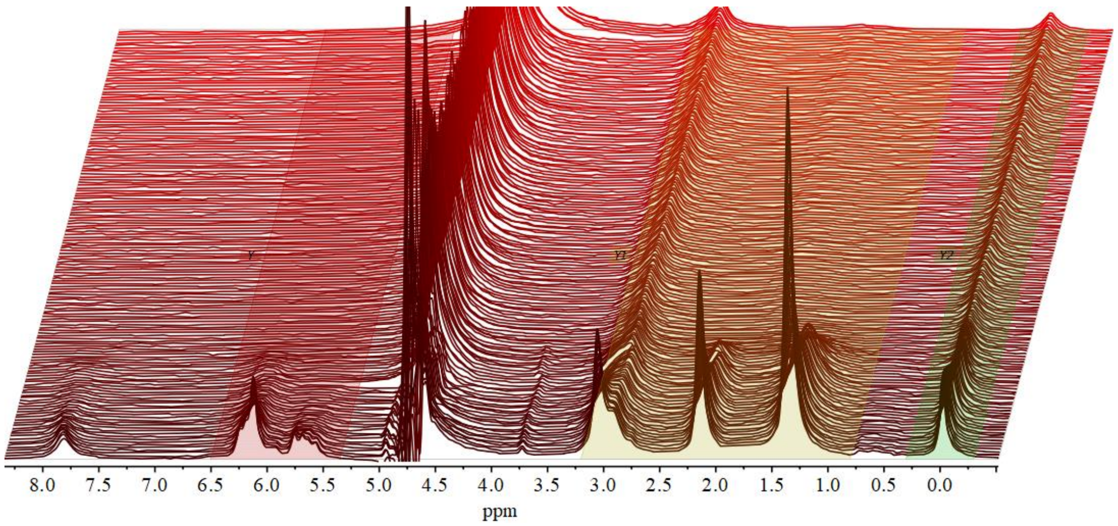

Figure S 4. Transient kinetic study ^{1}H NMR spectra during the synthesis targeting PDMAm$_{98}$-PDAAm$_{400}$ demonstrating the decrease in vinyl region between 5.50-6.50 ppm which is utilised to determine conversion.

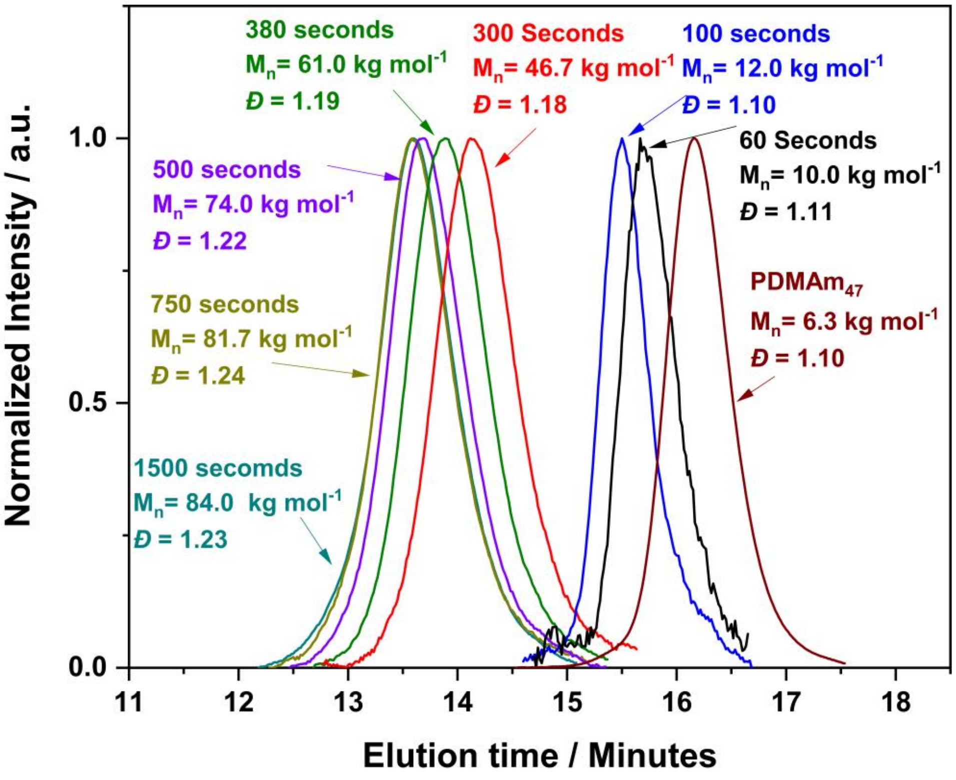

Figure S 5. GPC chromatograms of the chain extension during RAFT polymerisation targeting $PDMAm_{47}$-$PDAAm_{400}$ including $PDMAm_{47}$ macro-CTA and residence times varying between 60 and 1500 seconds. Systematic shift to the lower retention time as residence time increased indicate a growth in PDAAm block length until 750 seconds after which there is negligible growth with increased residence time.

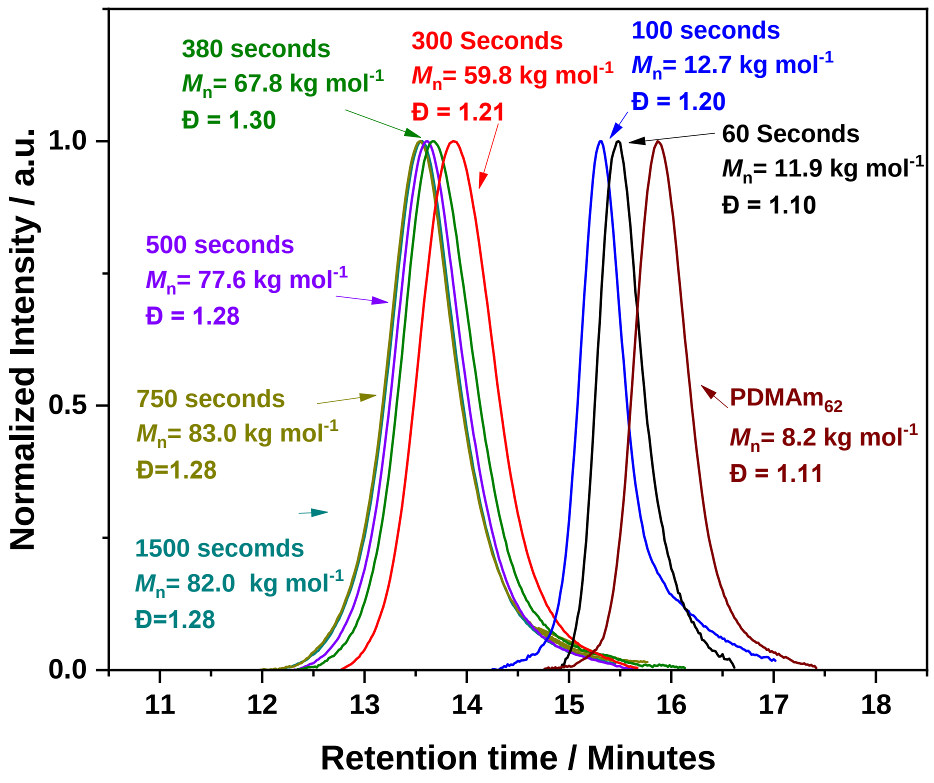

Figure S 6. GPC chromatograms of the chain extension during RAFT polymerisation targeting $PDMAm_{62}$-$PDAAm_{400}$ including $PDMAm_{62}$ macro-CTA and residence times varying between 60 and 1500 seconds. Systematic shift to the lower retention time as residence time increased indicate a growth in PDAAm block length until 750 seconds after which there is negligible growth with increased residence time.

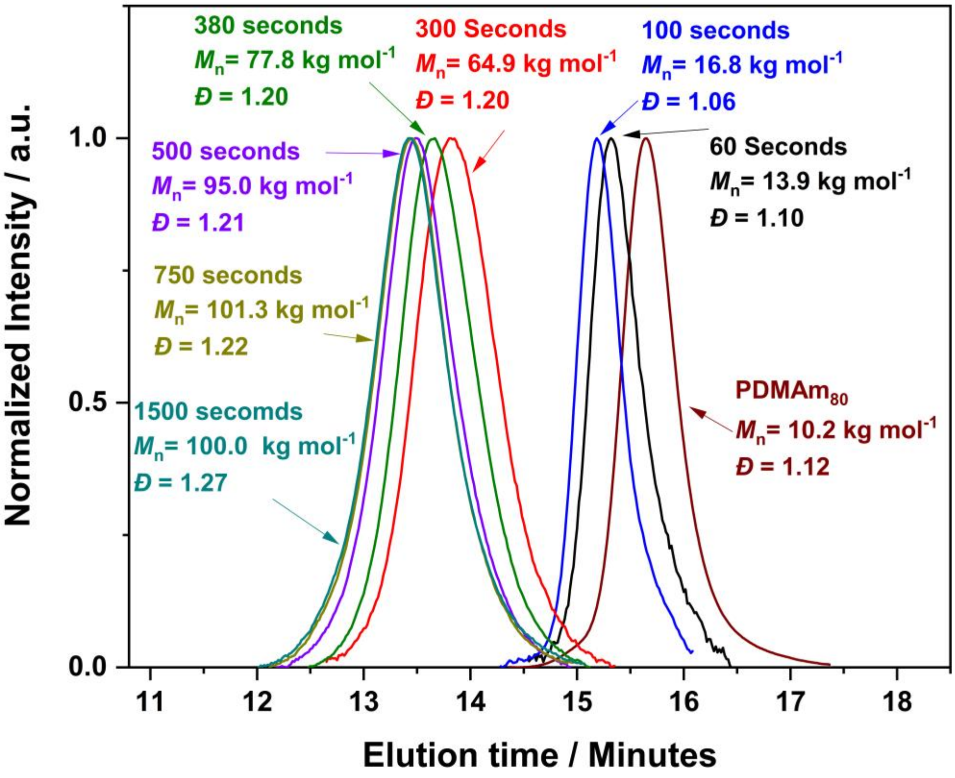

Figure S 7. GPC chromatograms of the chain extension during RAFT polymerisation targeting $PDMAm_{80}$-$PDAAm_{400}$ including $PDMAm_{80}$ macro-CTA and residence times varying between 60 and 1500 seconds. Systematic shift to the lower retention time as residence time increased indicate a growth in PDAAm block length until 750 seconds after which there is negligible growth with increased residence time.

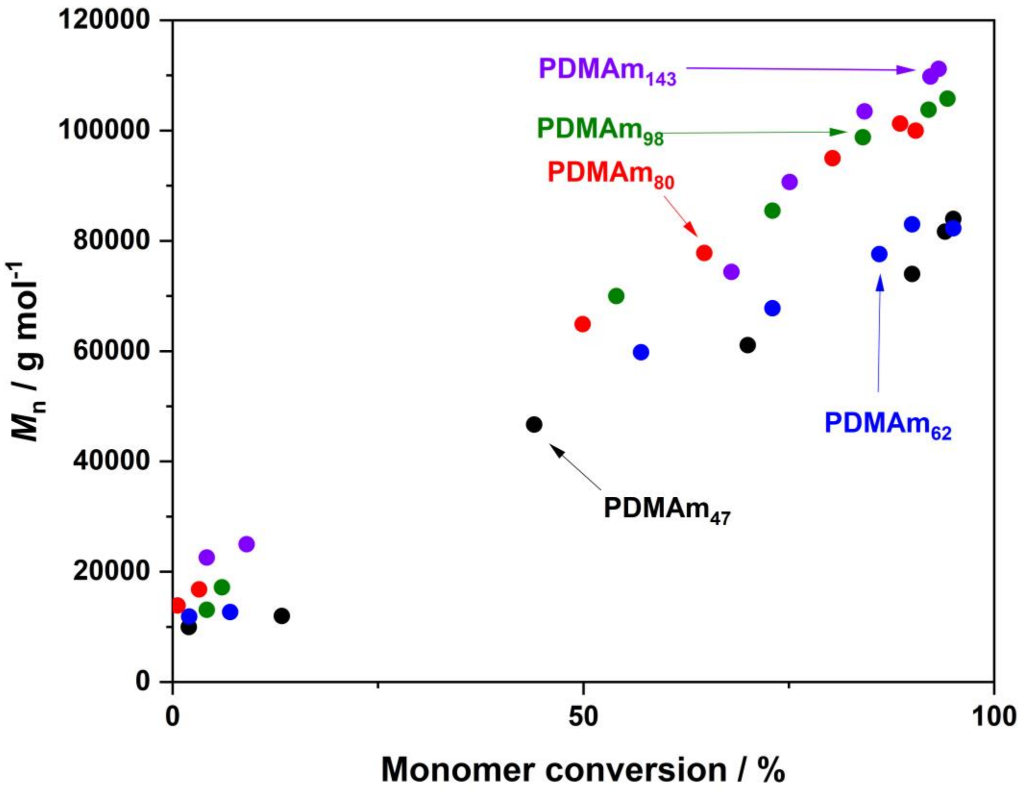

Figure S 8. The linear evolution of number average molecular weight (M_n) with monomer conversion when targeting the syntheses of PDMAm$_x$-PDAAm$_{400}$ indicating controlled polymerisation was maintained throughout the reaction.

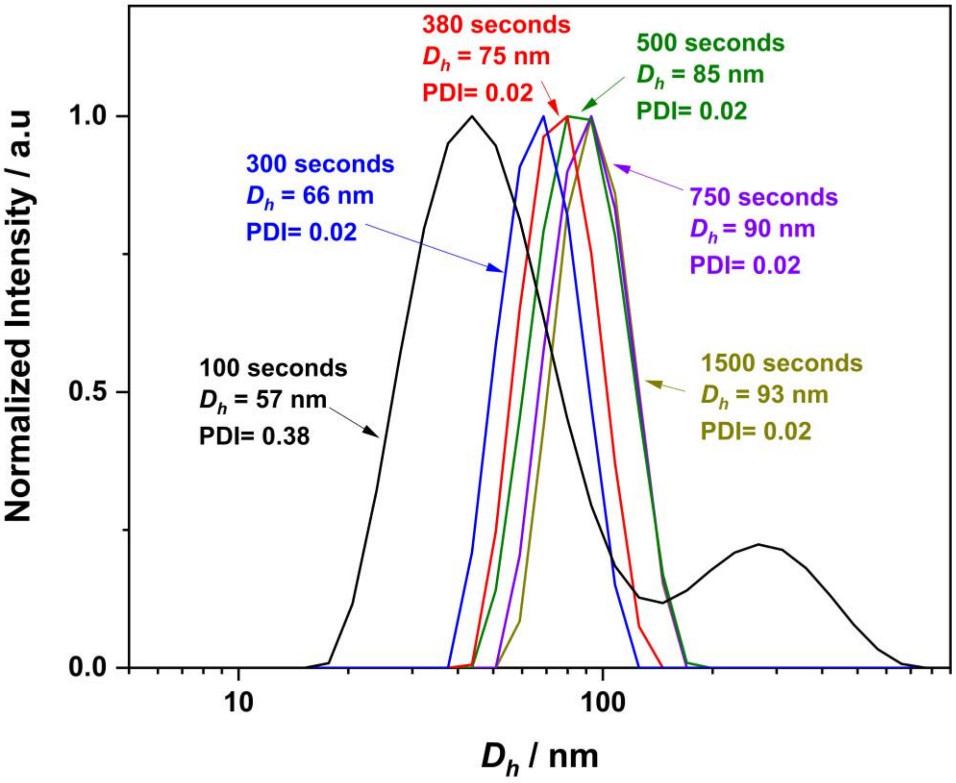

Figure S 9. DLS traces demonstrating particle growth during $PDMAm_{80}$-$PDAAm_{400}$ RAFT dispersion polymerisation at different residence times. Particles were found to have formed at 100 seconds but are highly disperse and a bimodal peak compared to higher residence times (PDI 0.38 compared to 0.02 thereafter). Significant particle growth is observed with an increase in residence time until 750 seconds minutes in which further increase results in minimal particle growth, indicating no further conversion had occurred. Intensity has been normalised to aid in comparison.

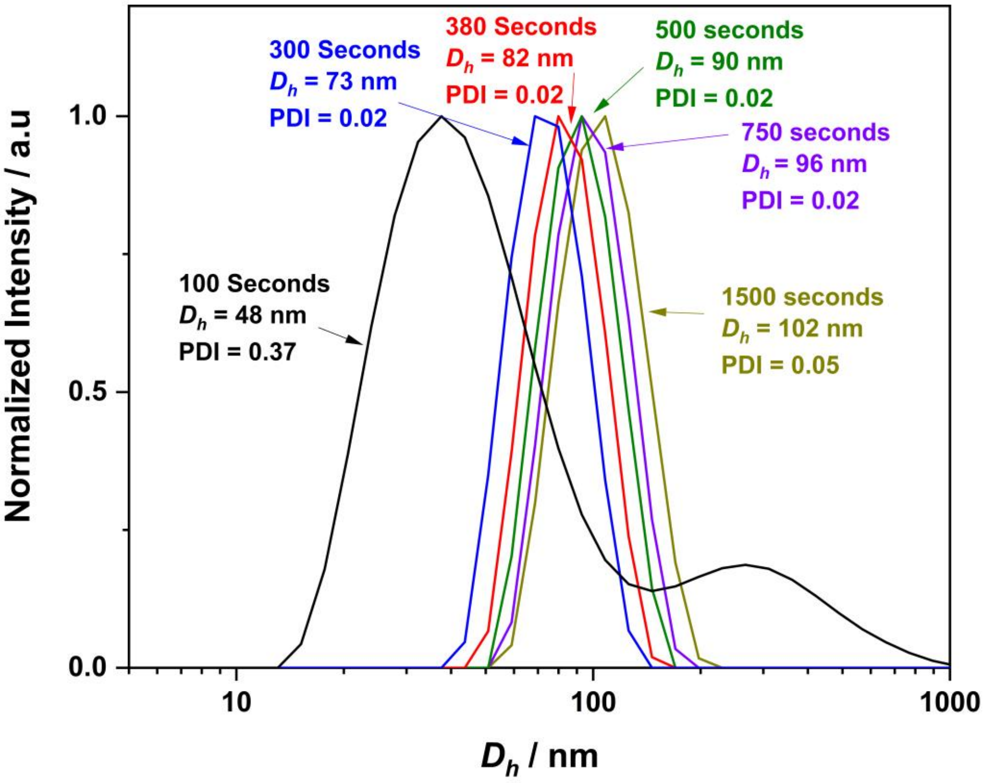

Figure S 10. DLS traces demonstrating particle growth during $PDMAm_{62}$-$PDAAm_{400}$ RAFT dispersion polymerisation at different residence times. Particles were found to have formed at 100 seconds but are highly disperse and a bimodal peak compared to higher residence times (PDI 0.78 compared to 0.02 thereafter). Significant particle growth is observed with an increase in residence time until 750 seconds in which further increase results in minimal particle growth, indicating no further conversion had occurred. Intensity has been normalised to aid in comparison.

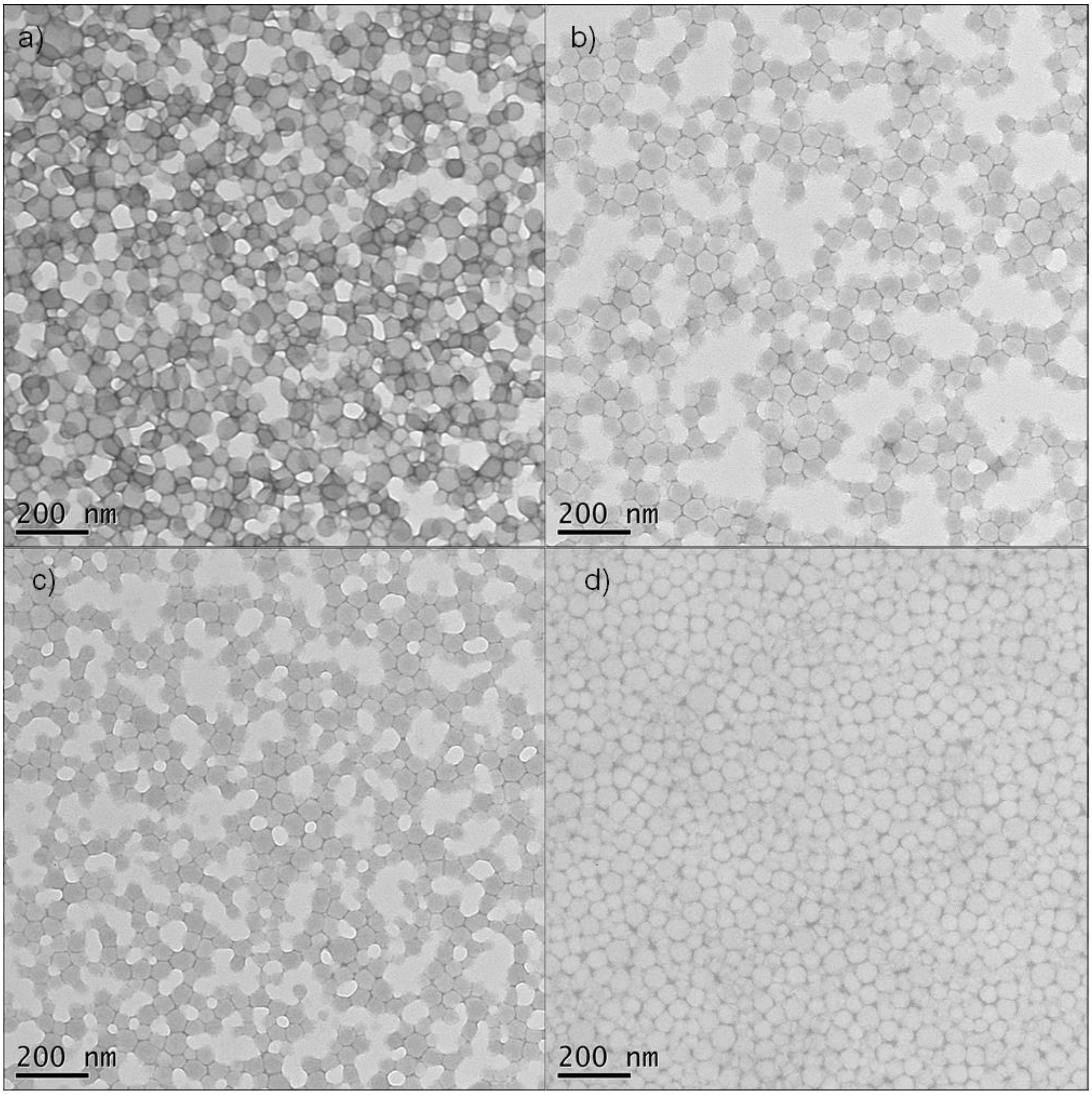

Figure S 11. TEM images of the spherical polymer nanoparticles formed at residence times during the synthesis targeting $PDMAm_{98}$-$PDAAm_{400}$ of **a)** 1500 seconds, **b)** 750 seconds, **c)** 500 seconds, **d)** 380 seconds. In all cases the resulting nanoparticles were spherical as indicated by the SAXS patterns.

SAXS Fitting

The SAXS patterns were fit to a spherical micelle model combined with a structure factor based upon the corresponding Percus-Yevick hard sphere model.[1,2] This approach is analogous to that taken by Derry et al who describe it in detail including the adaption of the original Spherical micelle model reported by Pedersen.[3–5] The individual scattering patterns were fitted using the Irena fitting Package within Igor Pro v8.[1,6]

X-ray scattering length densities (ξ) were calculated in Irena SAXS macro where: ξ_{PDAAm} = 10.43 x 10^{10} cm^{-2} ξ_{PDMAm} = 10.72 x 10^{10} cm^{-2}), ξ_{H2O} = 9.43 x 10^{10} cm^{-2}. he R_g was initially fixed based upon theoretical values outlined by Byard *et.al.*[7] since the limitations of the q-range and lower flux failed to gain realistic values from fitting and the previous study showing little deviation from the theoretical value. However after refitting all SAXS patterns with a non-set R_g it was found to have little influence on the core radius and hence the non-set R_g values have been reported.

Calculation of aggregation number

Approximation of the number of chains within the core was calculated by (Eq. S1)

$$N_{agg} = (1 - x_{sol}) \times (\frac{\frac{4}{3}\pi R_c{}^3)}{V_c}) \qquad \text{(Eq. S1)}$$

In which N_{agg} is the number of chains per micelle, x_{sol} is the volume fraction of solvent within the core, R_c is the radius of the core and V_c is the volume of the core block calculated

by V_c is the volume of the core and corona polymer blocks which can be approximated by $V=(M_n \text{ polymer})/(N_A\rho)$ with the densities of PDMAm and PDAAm being (1.13 g cm^{-3}) and (1.16 g cm^{-3}.

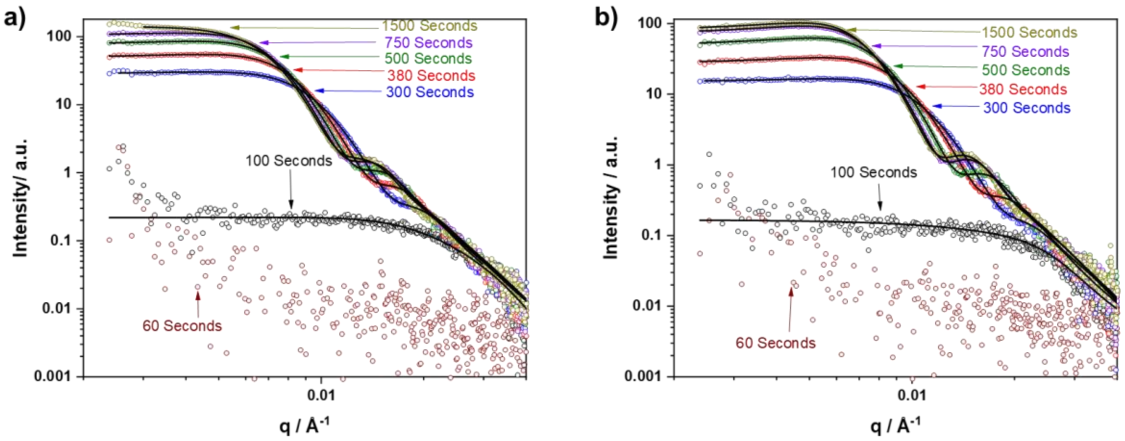

Figure S 12. SAXS scattering patterns obtained during the synthesis targeting **(a)** $PDMAm_{62}$-$PDAAm_{400}$ **(b)** $PDMAm_{80}$-$PDAAm_{400}$ within a tubular reactor at different residence times. Experimentally obtained scattering pattern (dots) is shown with model fitting (line*)*.

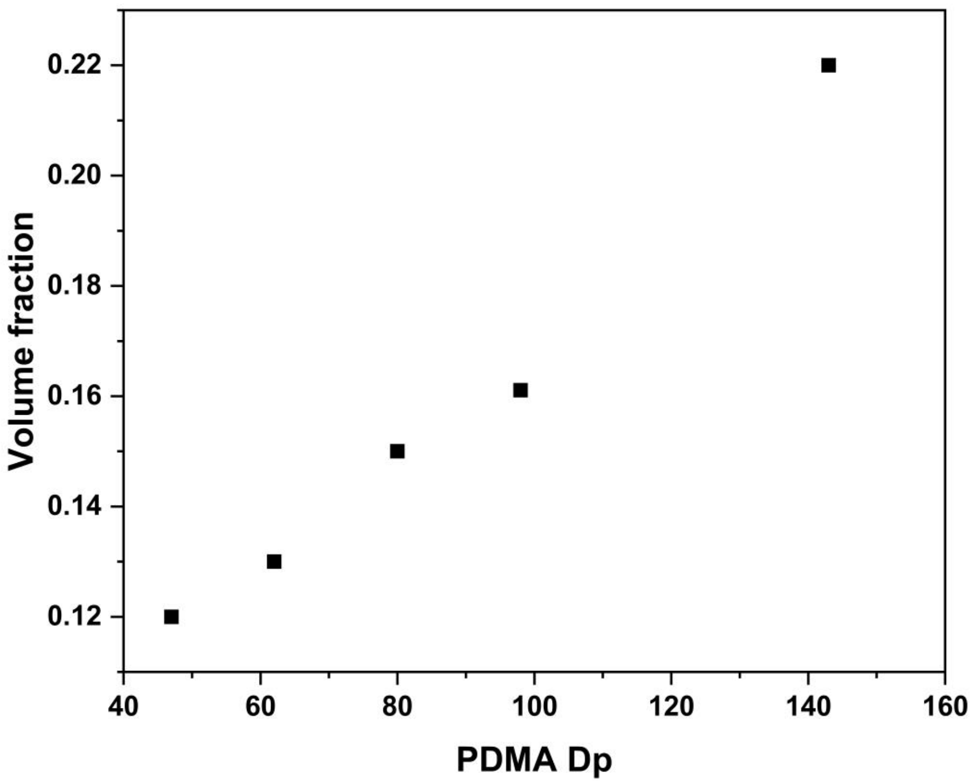

Figure S 13. The linear correlation between the volume fraction obtained from fitting SAXS patterns obtained at a residence times of 1500 seconds to spherical micelle SAXS models for $PDMAm_x$ in which x is 47,62,80,98,143 when targeting $PDMAm_x$-$PDAAm_{400}$.

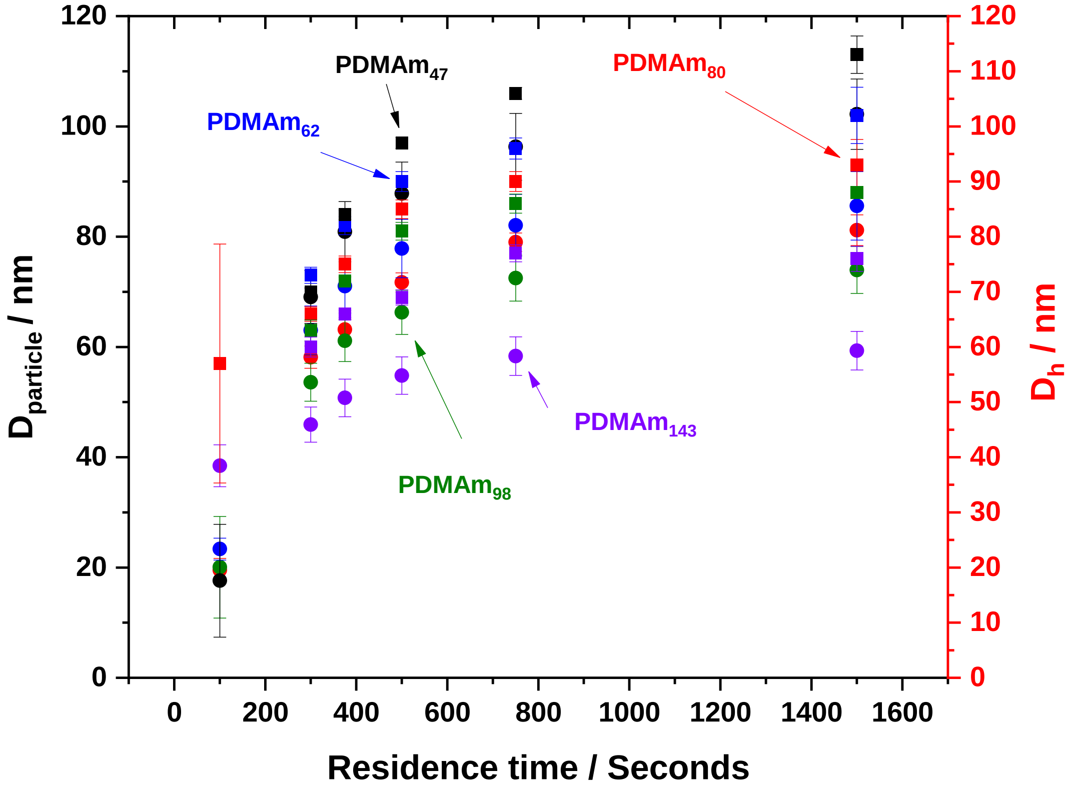

Figure S 14. Increase in D_{Particle} (square) and SAXS determined D_{Particle} (circle) with residence time when targeting $PDMAm_x$-$PDAAm_{400}$ with residence time in which X = 47,62,80,98 and 143. Error bars indicate standard deviation of the core size in the case of SAXS determined particle size and polydispersity for D_h.

References

1 S.R.Kline, Reduction and analysis of SANS and USANS data using IGOR Pro, *J.Appl.Cryst*, 2006, **39**, 895–900.

2 J. K. Percus and G. J. Yevick, Analysis of Classical Statistical Mechanics by Means of Collective Coordinates, *Phys. Rev.*, 1958, **110**, 1–13.

3 M. J. Derry, L. A. Fielding, N. J. Warren, C. J. Mable, A. J. Smith, O. O. Mykhaylyk and S. P. Armes, In situ small-angle X-ray scattering studies of sterically-stabilized diblock copolymer nanoparticles formed during polymerization-induced self-assembly in non-polar media, *Chem. Sci.*, 2016, **7**, 5078–590.

4 J. S. Pedersen, C. Svaneborg, K. Almdal, I. W. Hamley and R. N. Young, A small-angle neutron and x-ray contrast variation scattering study of the structure of block copolymer micelles: Corona shape and excluded volume interactions, *Macromolecules*, 2003, **36**, 416–433.

5 J. S. Pedersen and M. C. Gerstenberg, The structure of P85 Pluronic block copolymer micelles determined by small-angle neutron scattering, *Colloids Surfaces A Physicochem. Eng. Asp.*, 2003, **213**, 175–187.

6 J.Ilavsky and P.R.Jemian, Irena: tool suite for modeling and analysis of small-angle scattering, *J.Appl.Cryst*, 2009, **42**, 347–353.

7 S. J. Byard, C. T. O’Brien, M. J. Derry, M. Williams, O. O. Mykhaylyk, A. Blanazs and S. P. Armes, Unique aqueous self-assembly behavior of a thermoresponsive diblock copolymer, *Chem. Sci.*, 2020, **11**, 396–402.
